# Effects of wearable therapies on jump performance in sport horses

**DOI:** 10.3389/fvets.2023.1235932

**Published:** 2023-09-26

**Authors:** Therese E. Schmidt, Claire B. Gleason, Mercedez R. Samaniego, Robin R. White

**Affiliations:** School of Animal Sciences, Virginia Tech, Blacksburg, VA, United States

**Keywords:** Bayesian network, horses, jumping, performance, therapeutic boots

## Abstract

**Introduction:**

Failure to properly prepare the equine athlete for exercise and support post-exercise recovery is a contributing factor to physical breakdown and lameness. Equine physiotherapy was not introduced until the early twentieth century and has since evolved to allow for wearable therapies such as therapeutic boots to be accessible to a broad spectrum of equestrians. The purpose of this study was to evaluate the effects of ceramic boots, boots combining vibration and cryotherapy, and boots containing tourmaline on the performance of sport horses during jumping as well as to examine changes in vital signs in response to treatment.

**Methods:**

Eight healthy horses received the 3 therapeutic boot treatments or a control (no boot) in a Latin square experiment for a period of 5 days each. Horses performed approximately 10 min of exercise through a jump chute for the 5 consecutive days and jump performance parameters were recorded during each exercise session. Therapeutics were applied in the morning prior to exercise per the manufacturer’s recommendation and were removed only for exercise.

**Results and Discussion:**

In a Bayesian network analysis, changes in vital signs (heart rate, respiration, and temperature) were driven by individual animal, rather than boot treatment. Jump performance was influenced by boot treatment, physiological measurements, and individual animal. Therapeutic boots were associated with changes in conditional probabilities of numerous performance outcomes. This study indicates the use of wearable therapies may result in improved performance outcomes of sport horses in jumping exercises.

## 1. Introduction

Lameness is a significant cause of performance failures in sport horses that can lead to serious financial losses and compromised animal welfare (1, 2). Conformation, age, and physical exertion are a few of the many factors that influence a horse’s ability to remain in good physical health (3). Compromised physical health often presents as lameness, most frequently related to ailments in the distal limb. Disease and injury to the distal limb result in pain, decreased performance, and increased risk of reinjury. After clinical signs of these injuries present, veterinarians can treat these injuries with advanced or invasive therapies and prescribe rest to allow for the structure to heal under limited stress. Historically, therapeutic technologies were used for recovery following the occurrence of an injury. However, recent advancements in technology have simplified therapeutic devices which allows them to be commercially made, making them readily available to consumers to be intended for maintenance of the equine athlete.

Wearable therapeutics are used frequently by athletes, both human and equine, to prevent and protect from injury during exercise as well as to accelerate post-exercise recovery (4, 5). Many of these products contain materials like tourmaline or ceramic fibers that are infused into the fabric. Additional commercial products rely on mechanical stimulation to enhance therapeutic effects on tissues and may include cold therapy. Examples of each of these modes of action are available in commercial boots marketed for equines to be applied to the distal limb. Previous research has shown how these different technologies may have a positive influence on physical health. For example, tourmaline is a naturally occurring crystal that has been shown to enhance cellular metabolism by producing negative ions and converting electromagnetic waves into infrared radiation (6). The mechanism of this action is thought to be the improvement of vascular function (7). However, research on tourmaline’s effects in equines is lacking. Heat therapy is commonly used in horses (8). Materials that contain ceramics work to reflect heat back to the body to dilate vessels and increase blood flow (9). Alternatively, mechanical stimulation under the presence of ice or cold water improves circulation, increases the flow of cooled blood to efficiently cool down the horse post-exercise, and mitigates apoptosis in the soft tissue of the distal limb due to prolonged heat (8, 10). Although research has demonstrated the potential capacity for how each of these technologies may benefit physical health and recovery, information is lacking on how these technologies influence the horse in normal exercise conditions, free of clinical injury or recovery efforts. As such, there is a need to improve the characterization of how commercially available technologies perform when applied in a non-clinical setting to support regular exercise activities.

Examination of an athlete’s movement with the purpose of improving performance is a well-established element within various sports, including equestrian disciplines (11). By employing biomechanical inquiry, there exists the potential to objectively and quantitatively assess a horse’s movement when completing an exercise task in order to enhance the training program of that individual, inform orthopedic injury prevention efforts, or monitor certain aspects of the animal’s performance in that task (12, 13). A number of biomechanical measurements can be taken to describe a horse’s jump performance (12, 14). These may include the distance, height, and velocity achieved during the jump; the energy and force exerted; and the degree of balance that is maintained. Capturing these measurements provides the equestrian or investigator with an enhanced understanding of a horse’s completion of a jump, including assessment of the jumping shape achieved, termed the parabola or bascule (15). The Alogo Move Pro represents a wearable GPS-based sensor technology capable of tracking and recording jump performance measurements of interest (13). Internal measurement units such as the Alogo system possess several benefits over camera-utilizing optical motion capture systems, including cost, versatility, and ease of use in field settings (13), making them a valuable tool to leverage for capturing equine movement for training and research purposes.

The purpose of this study was to evaluate the effects of Lux Ceramic Therapy (Schneider Saddlery, Chagrin Falls, Ohio), Ice-Vibe (Horseware, Dundalk, Ireland), and Rambo Ionic (Horseware, Dundalk, Ireland) therapeutic boots on the performance of sport horses during jumping as well as monitor changes in vital signs as a response to treatment. We hypothesized that jumping performance and vitals would be altered in response to therapeutic boot application compared to receiving no boot.

## 2. Materials and methods

### 2.1. Animals and experimental design

All animal use and procedures described within this study were approved by the Virginia Tech Institutional Animal Care and Use Committee. Eight horses (3 thoroughbreds, 3 warmbloods, and 2 quarter horses; 2 mares and 6 geldings) in a light to moderate exercise program were used for data collection. Inclusion criteria were that horses had to be enrolled in the university’s equestrian lesson program and be determined clinically healthy by a veterinarian. Ages ranged from 9 to 22 years (mean = 15, median = 14.5). Body weights ranged from 448 to 577 kg (mean = 519, median = 522) and wither heights ranged from 143 to 173 cm (mean = 159, median = 159). Horses were stalled on weekdays and turned out into paddocks on weeknights and weekends. All horses were trained to canter through a jump chute off the left lead prior to the start of the study. Any horse refusing to acclimate to the jump chute was not selected for inclusion. Horse height and girth were measured for calibration of the Alogo Move Pro (Alogo Technologies, Vaud, Switzerland) stride sensor calculations (12, 13, 16). This system utilizes a GPS-based sensor to track the biomechanics of a moving horse and provide quantitative data on its jump performance (13).

The study utilized a Latin square design and consisted of 4 periods lasting 5 days each with a 10-day washout period in between. The use of a Latin square resulted in a sample size of 32 animal periods (8 horses × 4 periods). This sample size was deemed appropriate by an initial power analysis based on expected variability in the physiological and motion-based variables. Temperature, heart rate, and respiration rate were taken each morning prior to application of boots. Heart rates and respiration rates were taken manually using a stethoscope and stopwatch. Rectal temperatures were taken *via* digital thermometer. Two horses were assigned to each jump height (0.70 m, 0.80 m, 0.90 m, 1.0 m) according to athletic ability and treatment groups were arranged by jump height capability. Athletic ability was determined by the height for which they were used in the Virginia Tech lesson program, which is stratified based on the college equitation jump height divisions. Horses were jumped at the height for which they were used in lessons, which corresponded to a height where they reliably make a recognizable jumping effort, supporting the sensor in identifying the jump. The Latin square was used to designate a boot treatment for each animal for each period without repeating the treatment for the animal in another period. Treatments consisted of control (no boot), Rambo Ionic, Lux Ceramic Therapy, and Ice-Vibe boots.

### 2.2. Experimental procedure

For the 5 days of data collection during each period, each horse was brought in from the field at approximately 0730 h and placed into a 12 × 12 box stall. For animals receiving ceramic or ionic treatment during any given period, boots were applied as described previously immediately following arrival to the stall.

Beginning at 1200 h and running through roughly 1,500 h, the horses were individually exercised for approximately 10 min per day. Horses were not utilized for any other form of work or exercise (i.e., lessons) for the duration of the experiment. Preparation for exercise consisted of removing horses from the stalls, restraining them in cross ties, and removing boots (if applicable). Horses were outfitted with a lunging surcingle, equipped with the Alogo Move Pro sensor on the girth strap. To achieve balance, the girth was tightened evenly on each side. Horses were walked to the exercise arena where the sensor recording was started *via* Bluetooth connection using the Alogo app. Horses were warmed up on a lunge line in both directions at the walk, trot, and canter (approximately 3 min per gait), which is a standard practice at the equine center. Once acclimated, the horse was asked to canter 3 times through a jump chute with 3 obstacles set 7.3 m apart. The first obstacle in the chute was a single pole (0.15 m) to encourage the horse to enter the chute in a consistent stride. The jump chute design was structured to ensure that the horses encountered the second and third obstacles at a consistent stride to minimize variation in jump characteristics driven by poor striding. The final time through the jump chute, the 3rd jump in the sequence was raised 0.05 meters to the horse’s designated height. Jumping height, velocity, distance, energy, strike power, straightness, lateral balance, and longitudinal balance were recorded during this exercise (Table 1). Each day, horses were exercised in the order of lowest designated jumping height to highest designated jumping height to simplify jump readjustment for personnel. All warm-ups and exercise procedures were conducted without a rider.

**Table 1:** Definitions of jump performance parameters evaluated.

Parameter	Definition
Height	The height (m) that the horse achieves when going over the jump.
Velocity	The rate of propulsion (m/min) over a jump.
Distance	The distance (m) between the horse’s takeoff and landing points.
Desired bascule	The distance jumped is roughly four times the height jumped; the horse does not overjump nor does it hit the obstacle.
Energy	The energy (kJ) expended by the horse during the jump.
Strike power	The force (N) with which a horse’s hoof pushes against the ground when taking off for a jump.
Straightness	The horse’s uprightness (degrees) when jumping. Poor straightness would indicate a lean to the left or right.
Lateral balance	The horse’s distribution of weight between its left and right sides.
Longitudinal balance	The horse’s distribution of weight between fore and hind limbs; an indication of jump trajectory.
Efficiency	The horse jumps with above-average height but its energy expenditure and its strike power are below average.

After exercise, sensor recording was stopped before leaving the exercise arena. Once removed from the arena, horses were restrained in cross ties and lunging equipment was removed. Horses were lightly hosed over with cold water to remove sweat accumulation. Boots were then reapplied as previously described and horses were returned to their stalls. Designated boots were removed, and horses were turned out to their respective fields at 1750 h.

### 2.3. Treatment protocol

Lux Ceramic Therapy boots (Schneider Saddlery, Chagrin Falls, Ohio) were applied at 0800 h to the distal front limbs while the horse was stalled during the day per the manufacturer’s recommendation, with the exception that no adjustment period was granted in each period (i.e., wear time of the boot was not gradually increased). The boots were secured using Velcro attachments and were removed for exercise and turn out at 1730 h.

Horseware’s Ice-Vibe boots (Horseware Ireland, Dundalk, Ireland) were applied immediately before and after exercise. The boots have 3 proprietary vibration settings, but only the first 2 settings were used in this study per the manufacturer’s recommendation for use with exercise. Setting 1 was used prior to exercise for 10 min at a low frequency. After exercise, cold packs were placed underneath the Ice-Vibe boot and were worn for 20 min at Setting 2, a slightly higher frequency.

Rambo Ionic boots (Horseware, Dundalk, Ireland) were applied at 0800 h to the distal front limb while the horse was stalled during the day as per the manufacturer’s recommendation. Again, given the experimental conditions, no adjustment period was granted in each period. Two modes of application were used: a Velcro boot and a traditional standing wrap. The boots were only removed for exercise and turn out at 1730 h.

### 2.4. Statistical analysis

All statistical analyses were conducted using R (17). Results were analyzed as a linear mixed effects model using the lme4 package of R (18) where the fixed effects accounted for boot type and day of exposure, and the random effects accounted for period, animal, set jump height, and jump iteration. An analysis of variance using Satterthwaite’s method was performed to assess the response variables of interest: heart rate, respiration rate, body temperature, jumping height, velocity, distance, energy, strike power, straightness, lateral balance, and longitudinal balance. Data were calculated as percent change (increase or decrease) from observations on days 2 through 5 and day 1 measurements. This was done in attempt to isolate any changes that occurred during wear of the boot after the initial day.

Straightness and lateral balance measurements were taken as absolute values, since any deviation from 0 is undesirable. Data means separation were calculated using estimated marginal means and the Tukey method. Effects of treatment were evaluated based on boot type, day, and the interaction of boot type and day. Effects of day could not be provided for velocity or distance due to insufficient data. Statistical significance was considered when *p* < 0.05.

In consideration of the complex and interconnected nature of equine physiological and biomechanical responses, relationships between variables were explored *via* a Bayesian learning network (BLN). While a novel approach to this specific type of investigation in equines, Bayesian networks have been successfully applied in other specialties of animal and veterinary science, including nutrition (19), reproduction (20), lactation (21), genetics (22), and epidemiology (23, 24). Bayesian networks possess several advantages over traditional linear models for analyzing highly variable responses that are difficult to interpret in isolation. Firstly, the BLN considers all shifts in all measured variables simultaneously, conditional on the treatments that were applied to the individual subjects. It also allows incorporation of expert knowledge of the system so the analyst can indicate which variables are to respond to which other variables (25). An additional benefit is that the BLN assumes that dependencies exist among variables, and allows for explicit exploration of the quantitative associations of the responses in dependent variables. The result is a network fitted for the examination of relationships within a holistic representation of biology. The Bayesian model is not without its disadvantages, which include that it ignores some experimental effects (like period), it implies causality only through exploring the probability of the directionality of the relationship, and is unable to explore feedback loops, which might exist within this context (26, 27). Importantly, the network is not being used a prediction tool in this case, only as a strategy to understand the structure of relationships in the data in a manner more holistic than traditional statistical analysis.

The BLN was generated using the bnlearn R package (28) and a visual representation of the network was produced using the Rgraphviz package (29). The variables used included boot type; animal; vital signs (heat rate, respiration rate, and body temperature; jump performance parameters (height, velocity, distance, energy, strike power, straightness, lateral balance, and longitudinal balance); and standard deviations associated with the jump performance parameters. A directed acyclic graph and a score-based hill-climbing algorithm were employed in generation of the network. Any missing data was interpolated for by the network. Nodes represent the variables evaluated and edges represent the conditional dependencies among the variables. For example, an edge from node A to node B indicates that node A has a direct influence on node B. This relationship can be expressed as node B is conditionally dependent on node A. The BLN was used to generate conditional probability (CP) distributions for jumping performance outcomes for each individual horse to facilitate comparison between the average horse receiving a boot treatment and the average horse receiving the control. Change in CP was calculated as mean CP with boot divided by mean CP with no boot (control). In addition to the jump parameter means evaluated in the BLN, 2 new variables were computed: desired bascule and efficiency. Definitions of all jump parameters are given in Table 1.

## 3. Results and discussion

**Figure 1:**
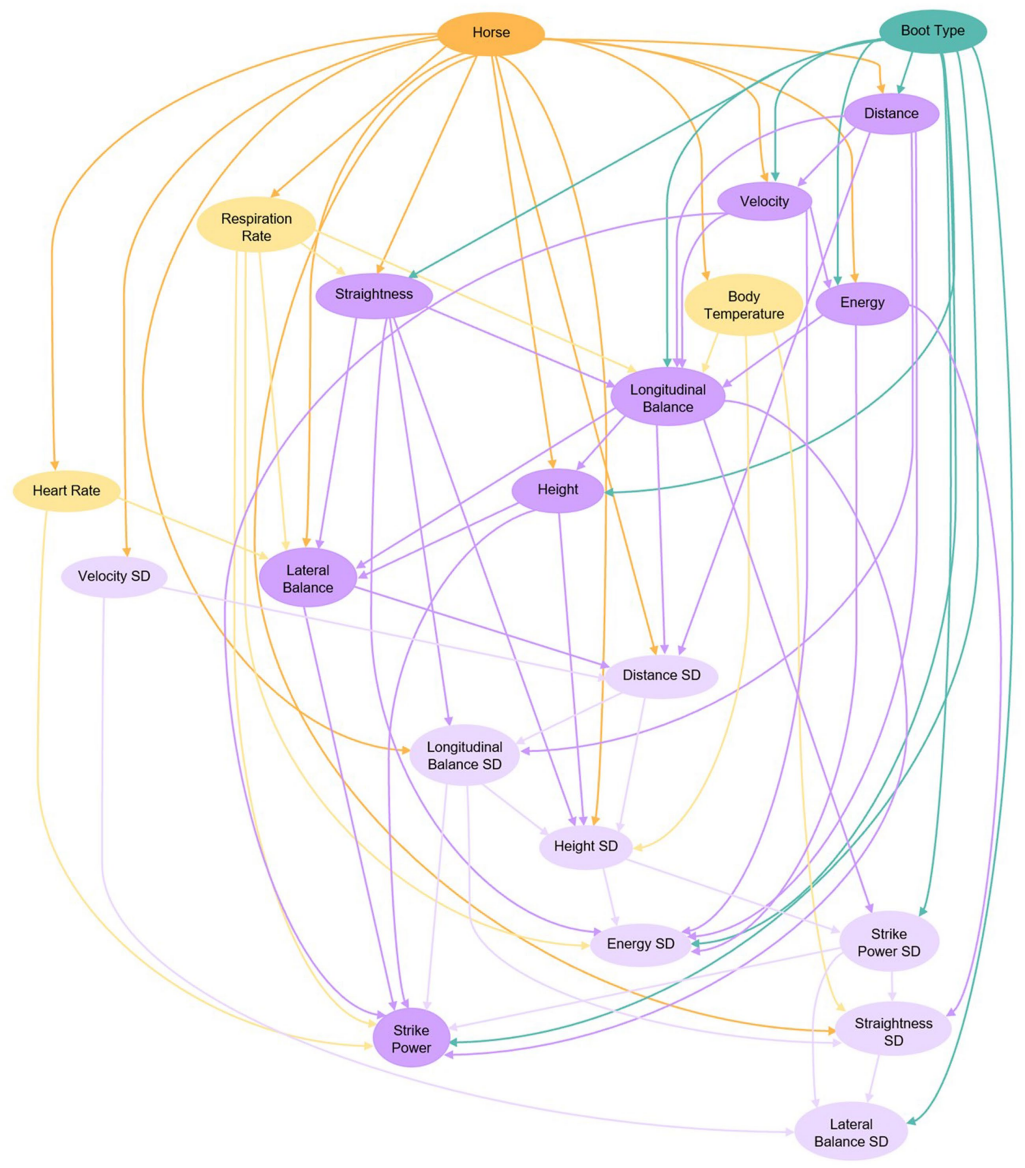
Bayesian learning network examining relationships associated with therapeutic boot type (green node), horse (orange node), vital signs (yellow nodes), jumping performance parameters (purple nodes), and variability in jump parameters (light purple nodes). Conditional dependencies are depicted by arrows such that the variable being pointed to is conditionally dependent upon the variable that the arrow originates from.

This study evaluated the impacts of employing 3 commercially available therapeutic equine boots on vital signs and jump performance parameters of sport horses. The design of this study allowed the horse to simulate a jumping exercise without rider intervention. With the influence of the rider, the horse may balance differently and compromise gait quality. The use of a BLN in the analysis of this data allowed for exploration of the structural associations within the data as a whole and allowed calculation of probabilities of performance success conditionally dependent on use of the individual therapeutic boots. Within the BLN, the major drivers of change were individual horse and boot type, as indicated by their high positioning in the network with numerous edges radiating from them (Figure 1). Strengths of relationships identified within the BLN are located in Supplementary Table S1 and a data summary is located in Supplementary Table S2. Individual horse was the primary driver of change, exerting influence on all physiological parameters and almost all jump variables. Between-animal variation is a frequent and well-established challenge for traditional research methods (30). The Bayesian approach allows examination of responses conditional on individual animal and treatment, which makes standardization within animal and within treatment possible. Treatment means and *p*-values are provided (Table 2) to maintain consistency with current literature practice. However, the BLN results will serve as the primary focus because this analytical approach is more appropriate for evaluating and presenting effects of the therapies within a complex ecosystem of responses that includes both physiological and biomechanical parameters.

**Table 2:** Estimated marginal means for treatment groups and *P*-values for the effects of boot treatment, day, and the interaction of boot treatment and day.

	Treatment means, % change				*p*-values^a,b^		
Response variable	Ceramic	Ice-Vibe	Ionic	Control	Boot	Day	Boot × day
Vital signs							
Heart rate	−1.70	8.98	1.64	−3.20	**<0.001**	0.98	0.45
Respiration rate	−9.07	−3.99	−1.88	−9.64	0.39	0.71	0.91
Body temperature	−0.103	0.000371	−0.0108	−0.0223	0.85	0.38	0.95
Jumping performance							
Height	3.01	3.64	−8.76	−2.36	**<0.001**	0.34	**0.026**
Velocity	243.3	53.4	46.5	−14.5	**<0.001**		
Distance	698.0	118.4	384.2	43.9	0.13		
Energy	235.3	78.7	140.7	44.2	**0.0048**		
Strike power	7.90	10.5	16.5	16.8	0.35	0.56	**<0.001**
Straightness	88.6	73.1	88.1	25.4	**<0.001**	**<0.001**	0.21
Lateral balance	−0.331	82.6	45.1	59.9	**0.004**	**0.0094**	**<0.001**
Longitudinal balance	−6.21	5.21	−8.22	−1.44	0.065	**<0.001**	0.11

^a^ Bolded values indicate significance. Significance was considered when *p* < 0.05.

^b^ Effects of day could not be calculated for velocity, distance, or energy due to insufficient data.

Data were calculated as percent change (increase or decrease) from observations on days 2 through 5 and day 1 measurements.

### 3.1. Influence on physiological parameters

Heart rate, respiration rate, and body temperature were not only monitored as an indicator of health, but also to determine response to treatment. These vital signs were evaluated as they are important for tracking fitness in the equine athlete (31). All vitals demonstrated conditional dependencies on individual horse but were not conditionally dependent on boot type (Figure 1). We therefore reject our hypothesis that boot application would be a driver of change in vital signs. Previous studies have reported high individual variation in equine vitals or cited it as a challenge in data analysis and interpretation (32–34). This is consistent with our observation that individual horse was the key driver of these variables. Considering it was not the intent of the study to generate a model or prediction tool, the results presented herein should not be misconstrued to represent all horses. The results are therefore representing the impact of boots on the animals used in the study. Traditional regression analysis indicated a boot effect (*p* < 0.001; Table 2) on heart rate, with Ice-Vibe boot use associated with an increase in heart rate. As described earlier, Ice-Vibe boot technology utilizes coolness and vibration. The other treatments do not employ these therapeutic measures. The Ice-Vibe treatment may have potentially resulted in greater positive change in resting heart rates if the unique sensations were raising the horses’ level of alertness. Studies evaluating whole-body vibration therapy in horses do not report consistent results with respect to change heart rates (35, 36). Previous research in humans has shown that whole-body vibration therapy increases heart rate and heart rate variability (37, 38). These mixed findings and the lack of comparable investigations using vibration solely on the distal equine limb indicate a need for further study to establish an effect on heart rate.

### 3.2. Influencers of jumping performance and conditional probabilities of performance outcomes

Within the Bayesian network, the jumping performance variables and their standard deviations appear to respond to individual animal, vital signs, and therapeutic boot type (Figure 1). This supports our hypothesis that performance variables would respond to boot treatment. The influence of horse on performance is logical as individual animals would not be expected to possess identical abilities with respect to an activity like jumping. The conditional dependencies of jump parameters on physiological variables would also be logical given their aforementioned importance in fitness and exercise. Jump parameters also demonstrated conditional dependencies on each other, which would be consistent with our understanding that these represent various measurements taken of the same physical body as it launches itself over an obstacle.

All jump performance variables possessed direct conditional dependencies on therapeutic boot use with the exception of lateral balance; however, lateral balance was linked to boot use through its conditional dependencies on height, straightness, and longitudinal balance. Linear regression indicated significant effects (*p* < 0.0049; Table 2) of boot type on jumping height, velocity, distance, energy, strike power, straightness, and lateral balance, in agreement with the Bayesian network. These results indicate that the various therapeutic technologies employed by the boots have the capacity to influence biomechanical responses in equine athletes during exercise.

Using the Bayesian network, changes in conditional probabilities of performance outcomes were calculated for each therapeutic boot type compared to the control of wearing no boot (Table 3). Horses receiving the Ceramic treatment were more likely to jump with greater velocity, strike power, and efficiency. While they were more likely to jump with less height and distance than when exposed to no boot, these horses were 5.79 times more likely to jump with a desired bascule, an indicator of appropriate relationship between height jumped and distance jumped. The Ceramic boot is intended to be thermotherapeutic, designed with ceramic fibers that reflect heat back to the limb as far infrared rays (FIR). Far infrared ray therapy has been demonstrated to increase blood circulation and tissue repair in humans (39, 40). There is also evidence that FIR can increase blood circulation independent of heat through a nitric oxide-related pathway (41). Despite the wide array of FIR therapeutic products and ceramic-related FIR therapeutic products currently marketed to horse owners, formal investigations of these therapies in horses appear to be unavailable in the literature. Nevertheless, heat therapy is an established treatment in equine veterinary medicine for stimulating local circulation, muscle relaxation, and tissue elasticity (42).

**Table 3:** Changes in conditional probabilities with respect to jump performance outcomes associated with therapeutic boot use relative to control^a^.

	Boot type		
Jump parameter	Ceramic	Ice-Vibe	Ionic
Height	0.337	1.27	0.952
Velocity	4.53	1.10	3.28
Distance	0.327	1.90	1.36
Desired bascule	5.79	1.60	0.734
Energy	0.769	0.351	0.468
Strike power	2.52	2.64	2.82
Straightness	0.754	1.48	0.386
Lateral balance	0.196	1.26	0.407
Longitudinal balance	0.184	1.31	1.03
Efficiency	1.87	0.991	0.891

a Conditional probability (CP) of change = mean CP with boot divided by mean CP with no boot (control).

Ice-Vibe treatment was associated with greater probability in increase of all jump parameters compared to the control, with the exception of energy and efficiency (Table 3). Like the Ceramic boot, the Ice-Vibe boot also employs thermotherapy, but uses cold instead of heat, termed cryotherapy. Cryotherapy is used in horses to reduce soreness and swelling from localized edema (42). The Ice-Vibe boot uses vibration therapy in addition to cryotherapy, which has been demonstrated to prevent soreness and improve muscle function in human patients (43, 44). In horses, investigations into the effects of vibration therapy appear to be mainly restricted to whole-body vibration (WBV) rather than localized vibration, which may not yield comparable results. However, one study conducted cycloidal vibration on the thoracic spine and individual muscles of horses, reporting increased range of motion of the thoracic spine and improved thoracic musculature (45). Despite having used the combined cooling and vibrating features of the Ice-Vibe boot (per the manufacturer’s recommendation) for this particular investigation in order to mimic the typical user setting, future work could test these cooling and vibrating effects individually for comparison, in addition to the combined effect.

Performance outcome probabilities associated with Ionic treatment included increases in velocity, distance, strike power, and longitudinal balance, and decreases in the remaining parameters compared to the control. The Ionic boot is advertised by the manufacturer as utilizing negative ions released by tourmaline to exert its therapeutic effect. Few scientific studies have been conducted on the benefits of negative ion technology and no available literature appears to involve equine subjects or exercise physiology. More appropriately controlled work is required to better ascertain how tourmaline’s effects may impact equine tissues and movement.

### 3.3. Limitations and future directions

Determining the exact mechanism or mechanisms by which the therapeutic boots altered likelihoods of performance outcomes was outside the scope of the current investigation, but could serve as a focus of future work. Evidence of the physiological effects of these therapies which would lead to altered performance is likely localized at the site of application. Future studies could employ doppler technology to evaluate blood flow changes in response to boot use and histological evaluation could determine if changes in tissue concentration of stress biomarkers are occurring.

Studies in humans have reported that the wearing of ankle weights may influence gait and physical fitness of patients (46, 47). The weights of the therapeutic boots evaluated in this investigation differed by approximately 25 percent but this difference was small in comparison to the body weight of horses. However, because they represent added weight compared to the control of wearing no boot, the existence of a weight effect independent of the specific therapeutic effect (thermal, vibrational, etc.) is unknown. Determining this was outside the scope of the present study, and further work is needed to compare the particular therapeutic action with a placebo boot of the same weight.

Horse activity and behavior were not monitored when horses were turned out in the pasture at nights and on weekends; however, significant voluntary energy expenditure when loose (i.e., galloping) was not expected to have occurred given the mature ages of our subjects (>8 years). Nevertheless, it may be advisable for future investigations to employ pedometers or other activity tracking technologies for turn-out periods when horses are not under observation to monitor between-animal variation in voluntary energy expenditure, especially if the subjects are young or particularly active.

The fact that the Alogo app was unable to calculate velocity and distance in some instances in this investigation indicates that it may occasionally have difficulty detecting jump efforts. Independent validation of the Alogo Move Pro system by Guyard et al. (13) reported its accuracy as 5.5 to 29.2 percent and precision as 2.8 to 18.2 percent for tracking jump parameters. The authors maintained this performance was acceptable under field study conditions and supported the use of this device for scientific publications. Nevertheless, continued improvement and development of this and similar technologies will help researchers to obtain higher quality data in a variety of experimental settings.

## 4. Conclusion

This paper evaluated Rambo Ionic, Lux Ceramic, and Ice-Vibe therapeutic boots on their abilities to influence vital signs and jump performance and parameters in sport horses. Physiological variables were influenced by individual animal but appeared to be independent of boot treatment. Jump performance parameters were found to respond to boot treatment and were also influenced by physiological measurements and individual animal. Boot use was associated with increased conditional probability of improved performance for a number of jump parameters. This investigation suggests the use of wearable therapeutics in a non-clinical setting may elicit beneficial performance responses in the equine athlete. Future research with these therapeutics should evaluate their performance in an environment that is representative of typical exercise between horse and rider.

## Data availability statement

The raw data supporting the conclusions of this article will be made available by the authors, without undue reservation.

## Ethics statement

The animal study was approved by the Virginia Tech Institutional Animal Care and Use Committee. The study was conducted in accordance with the local legislation and institutional requirements.

## Author contributions

TS was responsible for animal monitoring, data collection, data analysis, and manuscript preparation. CG was responsible for manuscript editing. MS assisted with animal monitoring and data collection. RW determined experimental design, conducted data analysis, and contributed to manuscript editing. All authors contributed to the article and approved the submitted version.

## Funding

This research was funded by the Virginia Tech Pat Stuart Experiential Learning Initiative.

## Conflict of interest

The authors declare that the research was conducted in the absence of any commercial or financial relationships that could be construed as a potential conflict of interest.

## Publisher’s note

All claims expressed in this article are solely those of the authors and do not necessarily represent those of their affiliated organizations, or those of the publisher, the editors and the reviewers. Any product that may be evaluated in this article, or claim that may be made by its manufacturer, is not guaranteed or endorsed by the publisher.
