# Supplementary material for: Effects of wearable therapies on jump performance in sport horses
Source: Front Vet Sci. 2023 Sep 26;10:1235932. doi: 10.3389/fvets.2023.1235932 (PMC10562572; doi:10.3389/fvets.2023.1235932)
Supplement: Supplementary file 1 [file Table_1.docx]

***Supplementary Material***

| **Supplementary Table S1.** Strengths of relationships identified within the Bayesian learning network | | |
| --- | --- | --- |
| Variable | Response | Strength |
| Horse | Heart rate | 0.97 |
| Horse | Respiration rate | 0.99 |
| Horse | Body temperature | 1.00 |
| Horse | Distance | 0.96 |
| Horse | Energy | 1.00 |
| Horse | Height | 1.00 |
| Horse | Lateral balance | 0.96 |
| Horse | Straightness | 0.94 |
| Horse | Velocity | 1.00 |
| Horse | Distance SD | 1.00 |
| Horse | Height SD | 0.92 |
| Horse | Longitudinal balance SD | 0.99 |
| Horse | Straightness SD | 0.90 |
| Horse | Velocity SD | 0.84 |
| Boot type | Distance | 0.88 |
| Boot type | Energy | 0.73 |
| Boot type | Height | 0.64 |
| Boot type | Longitudinal balance | 0.08 |
| Boot type | Straightness | 0.67 |
| Boot type | Strike power | 0.76 |
| Boot type | Velocity | 0.82 |
| Boot type | Energy SD | 0.58 |
| Boot type | Lateral balance SD | 0.65 |
| Boot type | Strike power SD | 0.76 |
| Heart rate | Lateral balance | 0.71 |
| Heart rate | Strike power | 0.21 |
| Respiration rate | Lateral balance | 0.55 |
| Respiration rate | Longitudinal balance | 0.27 |
| Respiration rate | Straightness | 0.31 |
| Respiration rate | Strike power | 0.47 |
| Respiration rate | Energy SD | 0.56 |
| Body temperature | Longitudinal balance | 0.13 |
| Body temperature | Height SD | 0.19 |
| Distance | Longitudinal balance | 0.49 |
| Distance | Velocity | 1.00 |
| Distance | Distance SD | 1.00 |
| Distance | Energy SD | 0.56 |
| Distance | Longitudinal balance SD | 0.88 |
| Energy | Longitudinal balance | 0.11 |
| Energy | Energy SD | 1.00 |
| Energy | Straightness SD | 0.25 |
| *(Continued)* | | |

| **Supplementary Table S1.**  Continued | | |
| --- | --- | --- |
| Variable | Response | Strength |
| Height | Lateral balance | 0.30 |
| Height | Strike power | 0.50 |
| Height | Height SD | 0.46 |
| Lateral balance | Strike power | 0.23 |
| Lateral balance | Distance SD | 0.43 |
| Longitudinal balance | Height | 0.88 |
| Longitudinal balance | Lateral balance | 0.41 |
| Longitudinal balance | Strike power | 0.92 |
| Longitudinal balance | Distance SD | 0.73 |
| Longitudinal balance | Strike power SD | 0.63 |
| Straightness | Lateral balance | 0.47 |
| Straightness | Longitudinal balance | 0.16 |
| Straightness | Energy SD | 0.48 |
| Straightness | Height SD | 0.23 |
| Straightness | Longitudinal balance SD | 0.49 |
| Velocity | Energy | 1.00 |
| Velocity | Longitudinal balance | 0.40 |
| Velocity | Strike power | 0.33 |
| Velocity | Energy SD | 0.58 |
| Distance SD | Height SD | 0.32 |
| Distance SD | Longitudinal balance SD | 0.55 |
| Height SD | Energy SD | 0.47 |
| Height SD | Strike power SD | 0.95 |
| Longitudinal balance SD | Strike power | 0.27 |
| Longitudinal balance SD | Height SD | 1.00 |
| Longitudinal balance SD | Straightness SD | 0.68 |
| Straightness SD | Lateral balance SD | 0.66 |
| Strike power SD | Strike power | 0.88 |
| Strike power SD | Lateral balance SD | 0.77 |
| Strike power SD | Straightness SD | 0.50 |
| Velocity SD | Distance SD | 0.96 |
| Velocity SD | Lateral balance SD | 0.95 |

| **Supplementary Table S2.** Data summary of variables | | | | |
| --- | --- | --- | --- | --- |
| Variable | Mean | SD | Minimum | Maximum |
| Vital signs |  |  |  |  |
| Heart rate, bpm | 39.5 | 4.7 | 28 | 52 |
| Respiration rate, brpm | 21.8 | 5.0 | 16 | 44 |
| Rectal temperature, °C | 98.8 | 0.88 | 96.1 | 100.2 |
| Jumping performance |  |  |  |  |
| Height, m | 1.01 | 0.24 | 0.0167 | 1.37 |
| Velocity, m/min | 272 | 139 | 40.0 | 766 |
| Distance, m | 3.55 | 1.8 | 0.530 | 9.43 |
| Energy, kJ | 1345 | 1001 | 4.02 | 3990 |
| Strike power, N | 4.01 | 1.5 | 1.00 | 13.9 |
| Straightness, deg | 1.85 | 0.83 | 0.00 | 5.00 |
| Lateral balance, deg | 6.24 | 4.9 | 0.00 | 26.8 |
| Longitudinal balance, deg | 24.1 | 9.1 | -14.7 | 57.9 |
